# PEEK/PEI Polymer Blends for Fused Filament Fabrication: Processing, Properties, and Printability

**DOI:** 10.3390/polym18010113

**Published:** 2025-12-30

**Authors:** Conor McCrickard, Adrian Boyd, Krzysztof Rodzen, Edward Archer, Faisal Manzoor, Jawad Ullah

**Affiliations:** School of Engineering, University of Ulster, York Street, Belfast BT15 1ED, UK

**Keywords:** polyether ether ketone, polyetherimide, 3D printing, crystallinity, mechanical properties

## Abstract

Printing with high-performance polymers such as polyether ether ketone (PEEK) and polyetherimide (PEI) presents issues regarding shrinkage and warpage due to elevated temperatures. One method highlighted to mitigate against this is through polymer blending. This study explores the development and characterization of PEEK and PEI blends as filament for fused filament fabrication (FFF) in additive manufacturing. Filaments were produced via melt extrusion using PEEK/PEI weight ratios 100/0, 80/20, 70/30, 60/40, 50/50, 40/60, 20/80, and 0/100 (wt.%). The aim is to identify an optimum blend which enhances printability and maintains mechanical and thermal integrity. The extruded filaments were first characterized through differential scanning calorimetry (DSC) to determine miscibility with all ratios presenting a single glass transition temperature. Samples were then 3D-printed and assessed through mechanical testing, DSC, X-ray diffraction (XRD), and scanning electron microscopy (SEM). The PEEK/PEI 80/20 (wt.%) blend was recognized as the optimum blend for maintaining crystallinity (35%) as well as good mechanical properties, averaging ultimate tensile strengths (UTSs) of 75.6 MPa and a Young’s modulus of 1338 MPa. Thermal properties also improved while warpage reduced and printability improved.

## 1. Introduction

Additive manufacturing (AM) or 3D printing encompasses a multitude of technologies that create three-dimensional objects from digital models through layer-by-layer deposition. AM boasts numerous advantages over traditional manufacturing techniques, such as design flexibility, reduction in material wastage, and reduced production time and cost [1]. New materials are constantly being developed, tailoring properties to specific applications to perform the desired function [2]. A plethora of additive manufacturing techniques are currently available, consisting of fused filament fabrication (FFF), electron beam melting (EBM), stereolithography (SLA), powder bed fusion, material jetting, selective laser sintering (SLS), sheet lamination, and binder jetting, all of which can be categorized into material extrusion, photopolymerization, sintering, and binding methods. Material extrusion looks at heating a material to a molten state through a nozzle before being deposited onto a bed. Photopolymerization involves the activation of a photopolymer resin, usually through light energy, so that it solidifies to create the desired geometries/design. Sintering looks at the use of a laser beam to selectively ‘sinter’ a material so that it solidifies, creating the object. The platform on which the powder is located then moves up by one layer thickness and the process is repeated. Similarly to sintering, binding follows an almost mirror procedure where only the powder is bonded together via droplets of a binding material which acts as an adhesive to join the powdered particles [2]. Fused filament fabrication (FFF) or fused deposition modelling (FDM) has received the greatest attention within research fields, which looks at the sequential addition of material layers according to a digital blueprint, which is traditionally carried out using thermoplastic materials as the feedstock [3]. Popular materials used within FFF include polylactic acid (PLA), acrylonitrile butadiene styrene (ABS), and polyethylene terephthalate glycol (PETG) [4]. High-performance polymers (HPPs) have become routinely used in AM in recent years, due to advances in the technology and the ability to print at elevated temperatures. This has led to the adoption of AM parts for tooling and end-use applications across industries including automotive, aerospace, and biomedical [5]. These HPPs present with properties which make them versatile due to exceptional mechanical strength, chemical resistance, and thermal stability, which promotes their integration across these high-demand industries [6].

Among these HPPs are polyether ether ketone (PEEK) and polyetherimide (PEI). PEEK is a member of the PAEK (Poly-Aryl-Ether-Ketone) family and is one of the most widely used polymers in engineering, possessing many excellent characteristics [7]. PEEK is categorized as a high-performing semi-crystalline polymer, consisting of both amorphous and crystalline regions. It has a high glass transition temperature of 143–148 °C and a high melting temperature of 300–340 °C [8]. Additionally, PEEK has become an important biomaterial as it is biocompatible and has been well-documented as a replacement for metals in orthopedic applications [9,10]. Polyetherimide (PEI), more commonly known by its commercial name Ultem, is an amorphous polymer which is more popular for its thermal properties and ability to perform in extreme heat conditions up to 200 °C [11]. PEI has a glass transition temperature of 217 °C [12]. The amorphous nature and presence of ether bonds allow for good flowability and lower viscosity, making PEI easier to process than PEEK while still offering high thermal and mechanical properties [13].

AM of HPP such as PEEK material can pose a number of challenges due to limitations associated with large thermal gradients, residual stress build-up, and interlayer adhesion, as well as the inability of printers to consistently maintain the required temperature [5]. The blending of PEEK and PEI has been described as a promising strategy for tailoring thermal, mechanical, and rheological properties, allowing for the combination of both material properties [14,15]. This blend has also been described as a way to mitigate limitations associated with printing HPP, such as warpage, by modifying crystallinity, lowering melt viscosity, and improving thermal homogenization [14]. This is brought about through the miscibility of the two polymers as it is one of the few miscible high-performance polymer blends, considering PEEK is semi-crystalline and PEI is amorphous [16,17,18].

This blend has been recognized as a promising material in the aerospace industry through the synergistic properties associated with the blend in terms of the mechanical strength and thermal stability required in high-temperature-dependent applications often associated with the aerospace environment [19]. Magri et al., in 2021, identified a 70/30 PEEK/PEI blend through DSC analysis for improved thermal and operating temperatures, as well as maintaining a degree of crystallinity. This work then explored preparation and characterization of the blend filament and the optimal printing parameters for the 70/30 blend [20,21]. Diouf–Lewis et al., in 2022, looked at adding 30 wt.% carbon fibre to a series of ratio blends for fused filament fabrication, concluding that the PEEK/PEI 80/20 blend with 30 wt.% of carbon fibre was optimal for maintaining strength and printability [19]. Diez–Pascual et al., in 2015, also identified the potentials of PEEK/PEI within a medical context and investigated blending these polymers and promoting bioactivity through addition of Nano-TiO_2_. A 70/30 PEEK/PEI was also selected as the matrix material, due to the balance between stiffness, toughness, thermal stability, and glass transition temperature. This work explored the blends processed through ultra-sonification and melt blending [22]. McCrickard et al., in 2025, highlighted the potentials of the blend in a medical context, highlighting the individual properties associated with each of the polymers, covering crystallinity, printing parameters, and reinforcements used [23].

Despite the increasing interest in PEEK and PEI for fused filament fabrication, most prior studies have focused on the individual polymers, reinforcement strategies, or processing optimization for a single composition [19,20,21,23]. Only a limited number of works have examined systematic variations in PEEK/PEI blend ratios and explored how PEI content specifically affects printability, crystallization behaviour, and resulting mechanical performance during FFF processing. To address these gaps, the present study investigates PEEK/PEI blends across a range of compositions with the aim of identifying an optimal formulation that enables improved processability while preserving desirable mechanical properties. Specifically, we examine the influence of PEI content on thermal behaviour and crystallization during FFF, providing insights that can support rational blend design for high-performance additive manufacturing applications.

## 2. Materials and Methods

The polyether ether ketone (PEEK) used was PEEK powder from Evonik (Vestakeep 2000FP, GmbH, Essen, Germany). A medium viscosity material with a density of 1.3 g/cm^3^ and a melt volume flow rate of 70 cm^3^/10 min was employed. The polyetherimide (PEI) was PEI powder from SABIC Innovative Plastics (Ultem 1010P200, Riyadh, Saudi Arabia) with a density of 1.27 g/cm^3^ and melt volume rate of 25 cm^3^/10 min at 360°/5 kg and 13 cm^3^/10 min at 340°/5 kg. The material was used in the form of powder. The powders were dried overnight in a circulating oven at 120 °C to remove any moisture before the extrusion process. Blends were prepared and mixed using manual mixing in a sealed container, at weight ratios of PEEK/PEI; 100/0, 80/20, 70/30, 60/40, 50/50, 40/60, 20/80, and 0/100. Table 1 presents the material information according to the technical data sheet (TDS) associated with the materials used and the SEM images of the raw powders.

### 2.1. Filament Preparation

**Figure 1:**
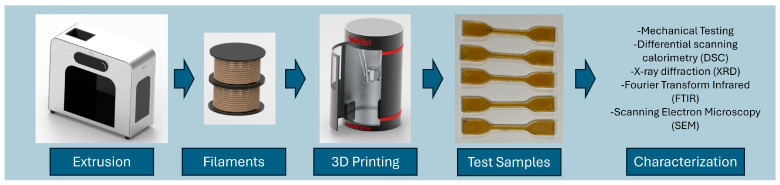
Flow chart illustrating the methodological process, from extrusion of filaments to the testing of print parts.

Filaments with a target diameter of 1.75 ± 0.05 mm were created using a commercial single-screw desktop extruder (3Devo Composer 450). To achieve a consistent diameter several parameters were carefully adjusted, including the feed rate, extrusion speed, temperature profile across the four heating zones, pulley speed, and fan speed. The dry powder blend was introduced via the hopper system. The process flow from extrusion, filament fabrication, to characterization is highlighted in Figure 1.

### 2.2. FTIR Analysis

Fourier transform infrared (FTIR) was performed using a Varian 640 IR spectrometer, with a diamond attenuated total reflectance (ATR) accessory. Samples were studied across a range of 400–4000 cm^−1^, at a resolution of 4 cm^−1^, with 20 scans per sample.

### 2.3. Three-Dimensional Printing of Samples

The G-code was generated using the CURA slicing software and used to produce 3D-printed samples using a commercial FFF printer SpiderBot 4.0 HT (Lugny, France). The printer consisted of an enclosed cylindrical chamber, six IR heaters capable of temperatures up to 450 °C, and a heated print bed reaching a maximum of 210 °C. Samples were produced according to ISO527-2 [24] type 5A using the blend filaments with the parameters displayed in Table 2.

### 2.4. DSC Analysis

Differential scanning calorimeter (DSC) was performed using a TA instrument DSC 250 (TA Instruments, New castle, DE, USA). Small samples of approx. A total of 10–15 mg was cut and used to obtain the thermogram of pure PEEK and PEI, and blends of PEEK/PEI filaments were produced via extrusion as well as 3D-printed samples. A heat–cool–heat run was deployed where samples were heated from 40 to 400 °C at a rate of 10 °C/min. Then, the samples were cooled at a rate of 10 °C/min to 25 °C before being reheated to 400 °C. The TRIOS software (TA Instruments) was then used to determine various thermal events, including glass transition temperature (Tg), melting temperature (Tm), cold crystallization (Tcc), crystallization (Tc), and the enthalpies for both melting and crystallization. The crystallinity (Xc) was calculated according to Equation (1):

(1) $$Xc\left(\%\right)=\frac{∆Hm-∆Hcc}{w\times ∆H{}^{\circ}m}\times 100$$

where ∆Hm is the enthalpy of melting; ∆Hcc is the enthalpy of cold crystallization; w is the weight fraction of PEEK in the blend; ∆H°m is the standard enthalpy of fusion for 100% crystalline PEEK, which was taken as 130 J/g. To assess the crystallinity for printed samples, the first heating run was used to gather information in relation to the different thermal events as a result of the printing process.

### 2.5. X-Ray Diffraction Analysis

X-ray diffraction (XRD) measurements were conducted, employing an Empyrean diffractometer (Malvern Panalytical, Almelo, The Netherlands) equipped with a Cu Kα radiation source (λ = 1.5406 Å) that was operated at 40 kV and 40 mA. Diffraction patterns were collected over a 2θ range of 5° to 90° with a step size of 0.04°.

Smoother portions of tensile samples were cut for analysis. Data analysis was performed using the Origin 2023b software. Crystallinity was qualitatively assessed by identifying characteristic PEEK peaks and changes in peak intensity as a function of PEI content. The degree of crystallinity was estimated by deconvoluting crystalline and amorphous peaks using baseline correction. The crystallinity was then estimated using Equation (2):

(2) $$Xc\left(\%\right)=\frac{Area\;of\;crystalline\;peaks}{Area\;of\;all\;peaks}\times 100$$

### 2.6. Mechanical Properties

Tensile strength and Young’s modulus of each ratio blend were measured using a universal testing machine, the Instron 5500R model (Instron, Norwood, MA, USA) tensile tester. Tensile testing was performed using a 5 KN load cell at a cross-hatch speed of 5 mm/min. The tensile strength and Young’s modulus were determined according to ISO527-2 using Type 5A samples at room temperature. For each ratio blend, five specimens were manufactured via 3D printing. To ensure warpage did not play a role in compromising mechanical properties, the area which would be clamped on the test samples was sanded to allow for a flatter surface, reserving the testing region.

### 2.7. Scanning Electron Microscopy (SEM)

Scanning electron microscopy (SEM) was performed using a Hitachi SU5000 field emission instrument (Oxford Instruments, Abingdon, UK). An acceleration voltage of 10 KeV under high vacuum was used during imaging. In order to make the surface of the samples conductive for SEM analysis to prevent charge build-up and improve image quality, the samples were sputter-coated with argon gas plasma using an Emitech K500X (Quorum Technologies, Laughton, UK). Coated samples were then mounted to a 51 mm diameter plate using carbon tape. Micrographs were captured at various magnifications.

## 3. Results and Discussion

### 3.1. DSC Results

The data gathered through DSC for the first heating of the neat PEEK, PEI, and blended filaments are displayed in Table 3. The miscibility of the two polymers within the extruded filament can be confirmed through the presence of a single glass transition temperature, which has been reported by multiple other researchers [18,25,26]. As the PEI content increases so does the glass transition temperature (Tg). The melting temperature (Tm) and crystallization temperature (Tc) also decrease as PEI concentration increases. It was noted during the first heating that blends 60/40, 50/50, 40/60, and 20/80 undergo cold crystallization by where the crystalline regions are allowed to form at the cold crystallization temperatures (Tcc). This is as a result of the rapid cooling during the extrusion process which could be exaggerated due to the use of the fans to cool the filament, allowing solidification at the desired filament diameter.

**Figure 2:**
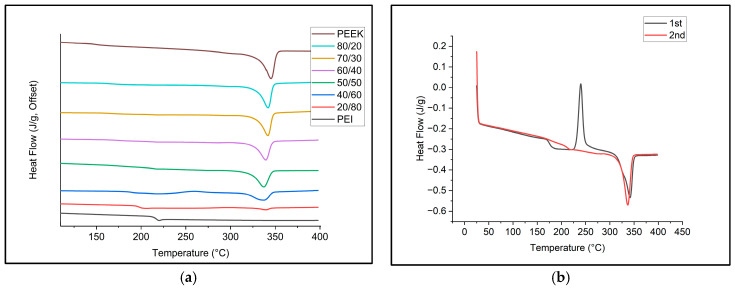
(**a**) Second heating of filament of PEEK/PEI. (**b**) First and second heating comparison of PEEK/PEI 5050.

During the cooling run, the filaments were cooled at a controlled rate of 10 °C/min which allows crystallization to take place. Crystallization temperature decreases as PEI concentration increases with the 20/80 blend behaving similarly to that of pure PEI; therefore, there is an absence of crystallization. Figure 2a presents the DSC results of the second heating curve of the samples. It can be observed that as a result of the cold crystallization during the cooling cycle, almost all blends show no cold crystallization peaks except 40/60 presenting a minor peak. The small peaks located around 200–220 °C for the PEI and PEEK/PEI 2080 blend represent the glass transition thermal event, while for other blends and neat PEEK, this thermal event is less even. A comparison of first and second heating curves for the 50/50 blend is shown in Figure 2b. It can be noted the first heating curve exhibits a cold crystallization peak which is no longer present in the second heating curve, following a cooling run. Ideally, replicating cooling conditions of the DSC during 3D printing would be the best for promoting maximum possible crystallization within the printed samples.

**Figure 3:**
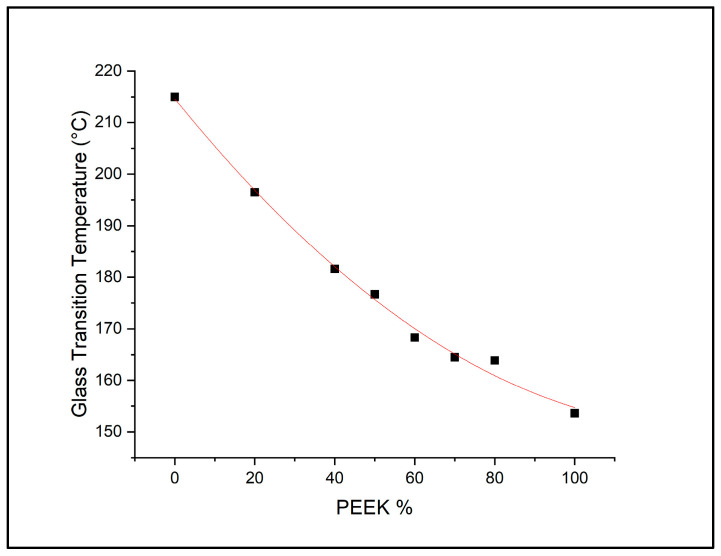
Glass transition temperatures of 3D-printed PEEK, PEI, and blends taken from first heating curve DSC analysis (Black squares represent the glass transitions temperatures, while the red line shows the fitted trendline).

Similarly to the DSC of the filaments, Figure 3 illustrates the printed samples which followed a similar trend with regards to increasing glass transition temperature (Tg) as PEI concentration increased, decreasing melting temperature (Tm) and crystallization temperature (Tc) with the increased PEI.

Table 4 presents DSC analysis of the 3D-printed samples which was taken from the first heating curve in order to determine the thermal events. The neat PEEK samples exhibited a distinct melting peak (Tm) at 324.9 °C and a crystallization peak (Tc) at 301.9 °C. PEEK exhibited a melting enthalpy of 41.8 J/g which is the largest across all blends, which is expected due to the well-defined crystalline regions associated with the semi-crystalline polymer. The 80/20 blend, however, presents with the highest crystallinity across all blends at a value of 35%. This can be attributed to the normalization of PEEK content, by using Equation (1) to calculate crystallinity, which takes into consideration PEEK content (0.8). Naturally, the melting enthalpy will be less than that of pure PEEK as there is less PEEK present. Magri et al., in 2021, concluded that the addition of PEI at this wt.% played the role of diluent layers and increased interlamination fluidity [20]. Along with PEEK, they are the only blends that do not possess a cold crystallization peak which can be as a result of numerous factors. In blends with higher PEEK content, PEI is unable to properly inhibit crystalline formation; therefore, the blends are able to reach much higher crystallinity values. The presence of the cold crystallization peak could be possibly due to the inability of the SpiderBot to maintain a high enough chamber temperature to slow the crystallization of the samples. It has been highlighted by multiple researchers that increasing chamber temperatures can enhance crystallization for pure PEEK [27]. It is expected that the same outcomes would be achieved for the blends. To test this, initial 70/30 samples were printed with lower chamber temperature using the integrated IR heaters and bed plate of the printer, at 410 °C and 150 °C, respectively. Blends of the 70/30 printed with lower thermal settings presented with a cold crystallization peak, indicating that the 3D-printing process did not allow for maximum crystallinity levels to be reached; once the chamber temperature was increased, cold crystallization peak became suppressed and crystallinity increased. Alongside the inability of the SpiderBot to reach elevated temperatures, the increasing presence of PEI across the blends also contributes to reduced crystallinity as PEI inhibits and restricts the movement of the crystalline regions following heating and cooling.

### 3.2. FTIR Results

**Figure 4:**
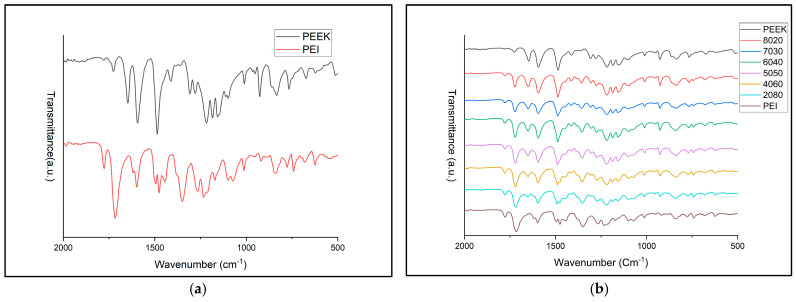
(**a**) The FTIR spectra of neat PEEK, PEI, and their blends. (**b**) FTIR spectra of PEEK and PEI, ranging from 2000 to 500 cm^−1^.

Fourier transform infrared spectroscopy was used in order to investigate the chemical structure of PEEK and PEI, as well as the various blends displayed in Figure 4a,b. The spectrum produced for neat PEEK filament is consistent with its aromatic and ether-based structure. The sharp peak located around 1650 cm^−1^ represents the ketone ring stretching (C=0) and peaks at 1593 cm^−1^ and 1487 cm^−1^ relate to the C=C stretching vibrations of the aromatic rings [28]. The spectra produced for PEI filament also produced noticeable peaks and bands which can be attributed to different functional groups. Peaks at 1780 cm^−1^ and 1720 cm^−1^ are present which are typical for imide carbonyl asymmetrical and symmetrical stretching. C-N stretching and bending can be linked to peaks at 1355 cm^−1^ and 743 cm^−1^ [29]. Throughout each of the blends, it is evident that as the PEI content increases, the peaks associated with the imide carbonyl at 1780 cm^−1^ and 1720 cm^−1^ grow more intense and become sharper when compared to neat PEEK. Similar patterns are recognized for growing PEEK content. The peak at 1650 cm^−1^ is initially well-defined for neat PEEK and gradually becomes suppressed as PEI increases. Through the comparison of the spectra for each of the blends, there is a clear overlap between the key features linked with the neat PEEK and PEI across all blends. Blends with higher PEI more closely relate to neat PEI; however, features of PEEK are still present, and the opposite is recognized for higher PEEK content.

### 3.3. Three Dimensional Printed Samples

**Figure 5:**
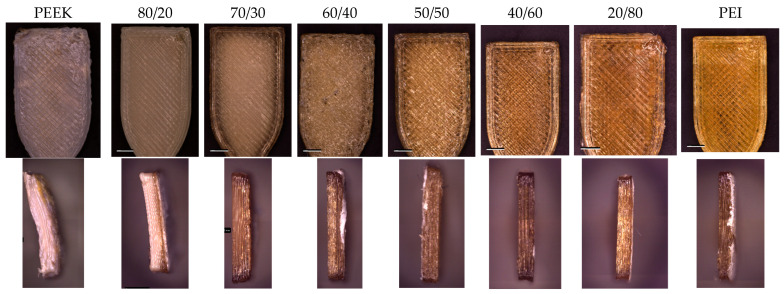
Images taken for PEEK, PEI, and blends using Zeiss Smartzoom (Carl Zeiss Microscopy GmbH, Oberkochen, Germany).

It was found across all ratios blends of PEEK/PEI that the printability and processability of the filament blends were massively improved in comparison to the neat PEEK and PEI. PEEK has a shrinkage rate between 1.2 and 1.5% which can be attributed to the densely packed crystalline regions becoming less ordered and then reordering during heating and cooling [30]. In semi-crystalline polymers with a high shrinkage rate, it can often be observed that more material is deposited on a previous layer that has decreased in size due to shrinkage. This phenomenon results in a considerable amount of warpage in prints, often compromising the dimensional stability of the printed samples. The addition of PEI to PEEK plays a role in restricting the mobility of the amorphous segments of PEEK, which acts as a way of limiting shrinkage and allowing for the printing of parts with higher dimensional stability [31]. Figure 5 shows images captured on the Zeiss smart zoom for PEEK, PEI, and their blends. The discrepancy is evident in the print quality of certain blends, with PEEK/PEI 80/20 and 40/60 exhibiting excellent print quality and minimized warpage. The observed quality of neat PEEK was extremely poor with a high degree of shrinkage and warpage. The surface image of PEEK shows the results of warpage and the impact it has on print quality as the nozzle came in contact with the surface, disrupting the printing pattern.

### 3.4. X-Ray Diffraction

**Figure 6:**
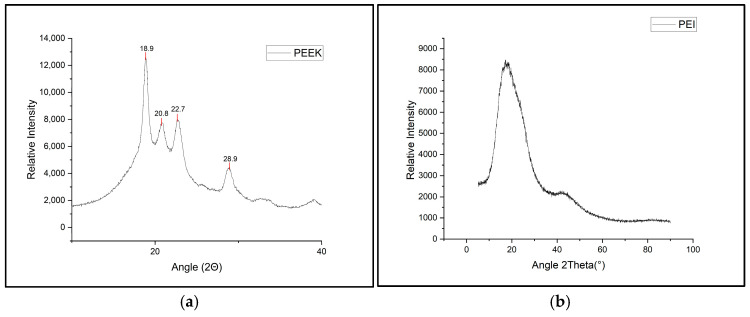
(**a**) XRD spectra showing peak location of 3D-printed neat PEEK. (**b**) XRD spectra of broad amorphous halo of 3D-printed PEI.

XRD was employed as a secondary method in order to investigate the crystallinity of 3D-printed samples and correlate the crystallinity results of XRD with the crystallinity observed during DSC analysis. Figure 6a shows neat PEEK which exhibited distinct diffraction peaks at 18.8°, 20.7°, 22.6°, and 28.9°, which closely matches the literature and is characteristic of its semi-crystalline nature [32]. PEI being amorphous exhibited no peaks and displayed a broad halo as seen in Figure 6b.

**Figure 7:**
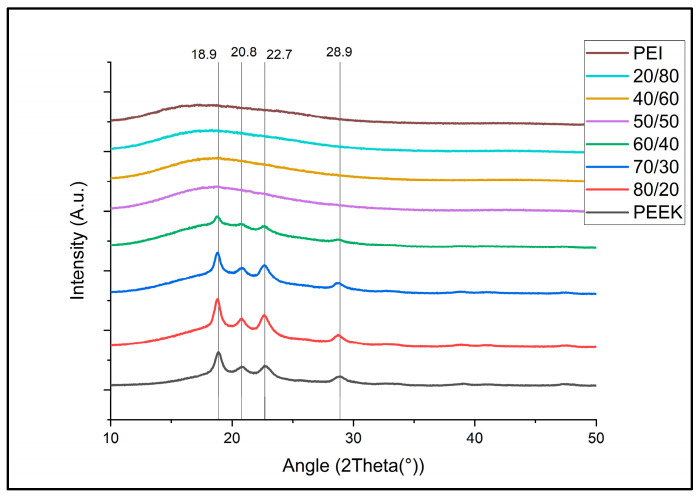
XRD comparison across all ratio blends with lines representing peak locations for neat PEEK.

Figure 7 shows the sharp peaks associated with PEEK crystalline regions; these peaks can also be recognized but gradually decrease and become suppressed for blends 80/20, 70/30, and 60/40, respectively, with the XRD pattern for 50/50 and below being almost identical to that of PEI. As the PEI content increases, the intensity of the peaks decreases, indicating that the crystalline regions are becoming suppressed.

**Figure 8:**
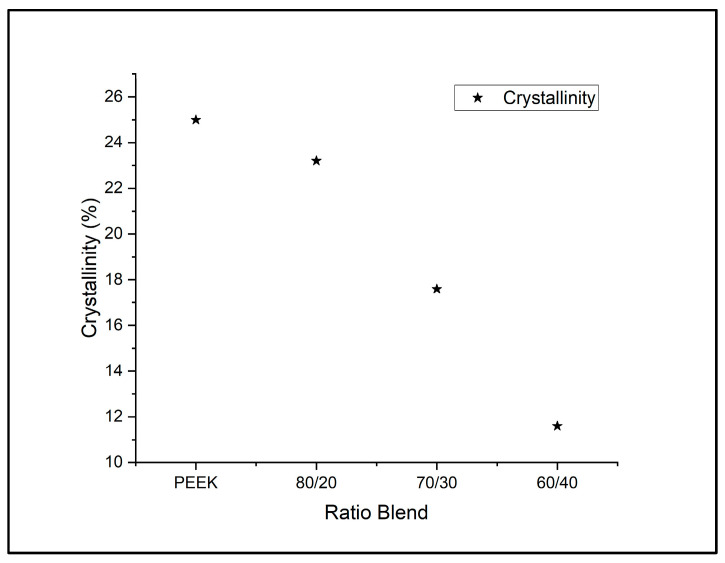
Crystallinity calculated through XRD analysis.

Doumeng et al., in 2021, recognized through a comparative study of XRD, DSC, and Raman spectroscopy that XRD tends to underestimate crystallinity and DSC can overestimate crystallinity for PEEK samples. This overestimation through DSC comes about through the competitive phenomenon of melting and crystallization taking place over a similar temperature range [33]. The over and underestimation can be observed through the comparison of the XRD results seen in Figure 8 and the DSC data of PEEK/PEI ratios in Table 4. DSC analysis of PEEK/PEI blends showed a progressive decrease in the melting enthalpy from 100% PEEK as PEI content increased, which indicates a reduction in crystallinity. Notably, samples even up to 40/60 PEEK/PEI had melting peaks, which suggests that crystallization is not entirely suppressed in this blend. By comparison with XRD patterns, there is a noticeable suppression of peaks as PEI content increases. This can be attributed to PEI acting as an inhibitor for the formation of the crystalline regions of PEEK [34]. Therefore, the crystal domains that do form are smaller, less ordered, and more constrained, leading to broader and less intense XRD peaks, indicating that although DSC confirms crystallinity even at lower PEEK contents, XRD reveals a significant reduction in the long-range order of PEEK crystals in the presence of PEI.

### 3.5. Mechanical Properties of 3D-Printed PEEK/PEI Blends

**Figure 9:**
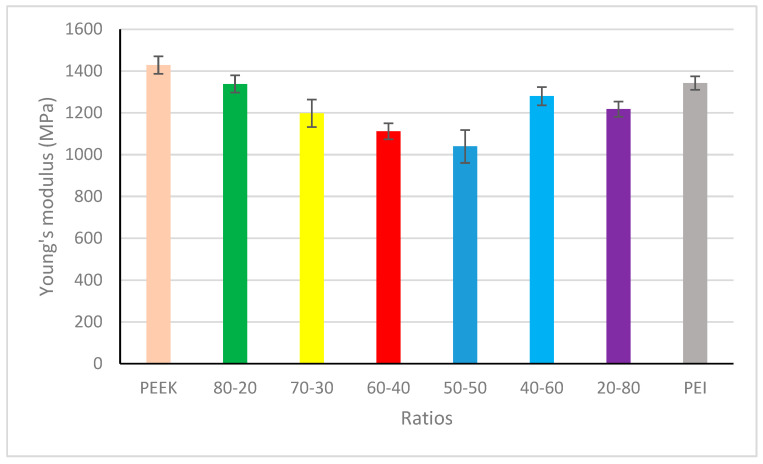
Young’s modulus for PEEK, PEI, and their blends.

The Young’s modulus of the 3D-printed samples is displayed in Figure 9 and Table 5 which follows a similar trend to those that have been previously reported through investigation of directly injected moulded PEEK, PEI, and their blends [15,26]. As expected through the anisotropic nature associated with FFF, the mechanical properties are slightly reduced when compared to injection-moulded samples. Arzak et al., in 1997, looked at a series of blends with differing contents of two high-performing polymers, making a comparison to the as-moulded samples and annealed samples. When as-moulded samples were mechanically tested, the Young’s modulus displayed an almost linear relationship with the value, increasing as the PEI concentration increased. When the samples were annealed at 185 °C for 24 h, this massively impacted the Young’s modulus of both neat materials and their blends. PEEK presented with higher modulus, decreasing towards the 50/50 blend before increasing again towards neat PEI [15]. By comparing the crystallization results with the mechanical properties, it can be concluded that the printing parameters and thermal conditions provide a means of allowing crystallization to take place in the blends with higher PEEK content, resulting in a higher Young’s modulus, and that the higher contents of PEI also contribute to higher modulus. The use of IR heaters of the SpiderBot has helped in slowing down the cooling process, replicating, to a lesser extent the effects of annealing. When printing semi-crystalline polymers such as PEEK, it is largely accepted that an increase in chamber temperature can result in higher crystallization by reaching higher temperatures [35]. We believe that due to the inability of the SpiderBot printer chamber to reach temperatures equivalent to that of the glass transition temperature of each of the blends, it has resulted in Young’s modulus values being in between fully amorphous prints and prints with maximum crystallinity.

**Figure 10:**
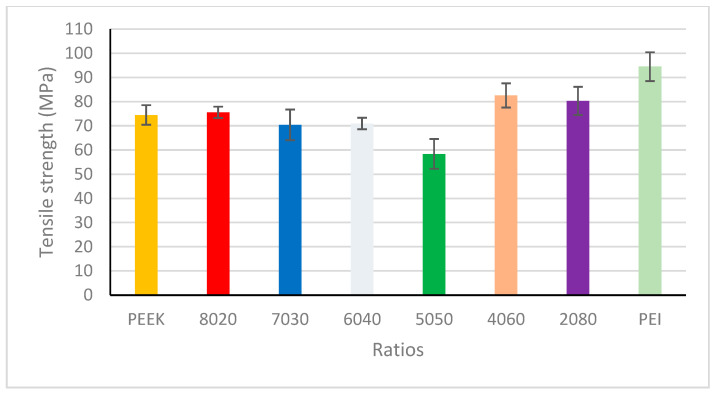
Tensile strengths of PEEK, PEI, and their blends.

**Figure 11:**
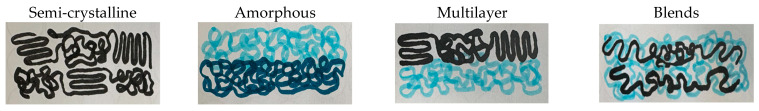
The arrangement of polymer chains in semi-crystalline, amorphous, multilayered, and blended polymers.

The tensile strengths across various compositions of PEEK/PEI are presented in Figure 10 and Table 5. Variables such as processing temperatures, composition ratio, and printing parameters exert an influence on crystallinity levels and mechanical properties. It is widely accepted that as crystallinity levels increase, so does the mechanical properties within crystalline samples [36]. Due to these crystalline regions in PEEK, it reduces the ability of chain diffusion and entanglement during the printing process, which reduces interlayer bonding strength and contributes to residual stress [37]. Amorphous polymers are less complicated in comparison to semi-crystalline polymers when it comes to printing. When the polymers are in an amorphous state, it allows for interdiffusion and entanglements across the bond interface [38]. Figure 11 provides a simplified illustration of polymer chain moments in semi-crystalline, amorphous, multilayered, and blended polymer prints in order to understand the tensile properties. PEEK/PEI multilayered prints offer a different alternative by where both polymers exist in separate phases joined by a homogenous miscible layer at the interlayer boundary [17]. The PEEK/PEI 40/60 and 20/80 blends present with the best tensile strengths across the blends with PEI possessing the highest tensile strength overall. It has been reported that the addition of PEI to PEEK can act as an inhibitor to the formation of the crystalline regions, hence reducing the crystallinity but allowing the means of better interdiffusion and entanglement similar to the behaviour of amorphous polymers. This process provides an explanation for the elevated tensile strengths.

The addition of 20 wt.% PEI to PEEK helps to increase crystallinity beyond the 33% value of neat PEEK to 35%. A similar trend was recognized by Magri et al., in 2021, who concluded that the addition of PEI played the role of diluent layers and increased the interlamination fluidity [20]. A study carried out by Magri et al. in 2021 identified 70/30 as the optimum blend following DSC analysis across 11 ratios and then printing at varying nozzle temperatures. Tensile strengths reached 78.6 MPa, which is slightly higher than the 70.4 MPa recorded during these tests. Three advantages of adding PEI to PEEK were highlighted, which included improved operating and service temperature by 20 °C, and decreased processing temperature while maintaining high tensile properties of PEEK [20]. Arzak et al., in 1997, also recognized a similar trend for injection-moulded PEEK/PEI blends in relation to the tensile strengths achieved. PEI has higher tensile strength than PEEK, and the addition of PEI to PEEK and the impact it has on tensile strength is counteracted by the subsequent reduction in crystallinity. This is further exaggerated at intermediate compositions [15].

### 3.6. SEM of Printed Samples

**Figure 12:**
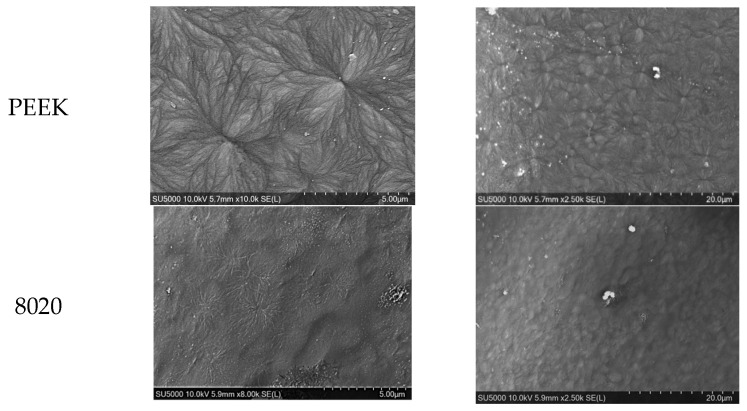

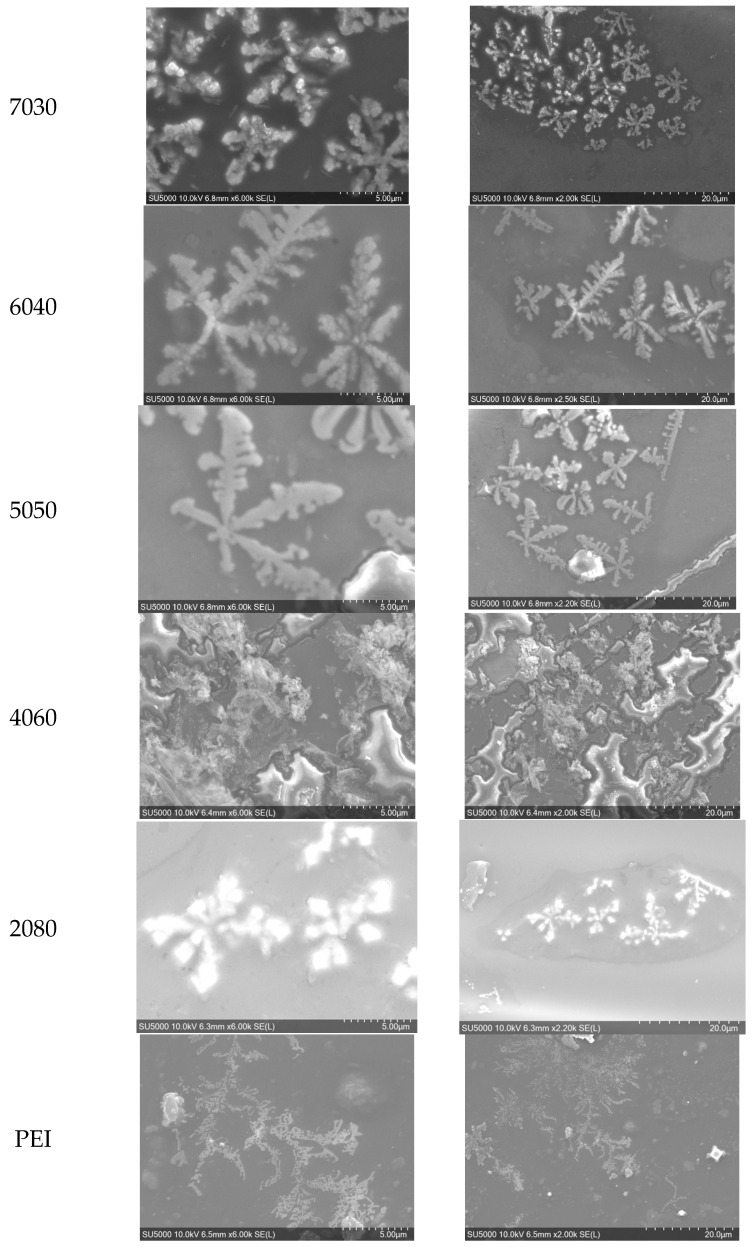
SEM images of the top surface of 3D-printed PEEK, PEI, and their blends (80/20, 70/30, 60/40, 50/50, 40/60, 20/80) at magnifications of 5 µm and 20 µm.

The morphology of the 3D-printed samples was characterized using SEM and presented in Figure 12 at magnifications of 5 and 20 µm. The SEM image of the 3D-printed neat PEEK and 80/20 PEEK/PEI blends demonstrates the presence of spherulitic structures. The presence of these structures can be related to favourable high-temperature crystallization conditions during the 3D-printing process [39]. Their presence also correlates with the DSC and XRD results as both presented the highest crystallinity of 32% and 35%, respectively. A clear difference in the visibility of the spherulite structures is noticeable between the PEEK and 80/20 blend. The spherulitic structure became less defined and much smaller in the 80/20 blend compared to neat PEEK. As the PEI content increases beyond 20% in the samples, no clear structures can be found in the SEM image. This is in agreement with the results of DSC and XRD data where higher crystallinities were observed. In the blends that contained PEI content higher than 20%, the SEM images displayed a frozen type of crystalline structure that resembled an almost snowflake appearance on the top surface of the 3D-printed samples, which is comparable to the structures observed by Rodzen et al. (2024) and Hudson et al. (1992), whereby crystallization has not been fully achieved [25,40]. This can be due to the crystalline regions of PEEK having reduced mobility to reform following melting and cooling as the PEI dilutes and freezes the mixture in an amorphous state, acting as an inhibitor for crystallinity [17].

## 4. Conclusions

Filaments were successfully extruded using a desktop extruder for PEEK, PEI, and a series of PEEK/PEI (80/20, 70/30, 60/40, 50/50, 40/60, 20/80) blends. The filaments were printed using FFF technology and were characterized using differential scanning calorimetry (DSC), X-ray diffraction (XRD), Fourier transform infrared spectroscopy (FTIR), scanning electron microscopy (SEM), and tensile testing. Through DSC, the miscibility of the filaments was confirmed by the presence of a single glass transition temperature. The miscibility of polymers is highly important, allowing the material of the filament to flow homogenously and uniformly through FFF. Miscibility was also confirmed through FTIR as all the blends presented with recognizable peaks and exhibited characteristic bands of both PEEK and PEI. The results from the XRD data correlate with the DSC. The DSC curves of PEEK and PEEK/PEI 80/20 exhibited the highest crystallinity and also displayed clear crystalline peaks in the XRD graphs. This is further strengthened through the presence of the spherulitic structures for neat PEEK and 80/20, while no clear structures were confirmed for other blends where PEI content surpasses 20 wt.%.

All blends were successfully printed using FFF. It can be concluded across all ratio blends that issues typically associated with the neat materials such as printability was improved and warpage was reduced with blended materials. PEEK/PEI 8020 was identified as the optimum blend across all ratios, maintaining a high degree of crystallinity at 35%. The blend also displayed good mechanical properties with the ultimate tensile strength reaching 75.6 MPa, which was beyond the neat PEEK and Young’s modulus value of 1338 MPa, which is slightly lower than PEEK. The addition of PEI to PEEK in the 8020 blend can provide a means of improving print quality; the properties associated with these high-performing polymers offer potentials beyond prototyping in the automotive, aerospace, and the biomedical industry.

The exploration of these PEEK/PEI blends for additive manufacturing offers a promising direction toward optimizing these high-performance polymers for advanced engineering applications. Through the combination of the superior mechanical properties of PEEK with the improved processability of PEI, these polymer blends have the potential to overcome the individual limitations of each.

## Figures and Tables

**Table 1:** PEEK and PEI material information and SEM images of the raw powders.

Polymer	Details	Image
Polyether Ether Ketone (PEEK)	Evonik (Vestakeep®2000FP, GmbH, Essen, Germany) Unreinforced, medium viscosity, fine powder material Density 1.3 g/cm^3^ Particle size 50 µm Melting temperature (Tm) = 340 °C	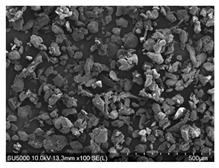
Polyetherimide (PEI)	SABIC Innovative Plastics (UltemTm 1010P200, Riyadh, Saudi Arabia) Unreinforced amorphous powder Density 1.27 g/cm^3^ Particle size 200–450 µm Glass transition temperature (Tg) = 217 °C	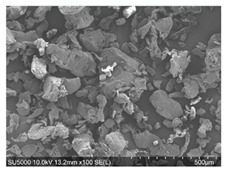

**Table 2:** Printing parameters used for 3D printing of PEEK, PEI, and their blends.

Print Parameter.	Value	Unit
Nozzle temperature	380	(°C)
Bedplate Temperature	180	(°C)
IR Heaters	450	(°C)
Print Speed	30	mm/s
Chamber Temperature	75	(°C)
Layer height	0.2	mm
Infill Density	100	%

**Table 3:** Thermal data from the first heating curve of filaments for PEEK, PEI, and their blends.

Blend	Glass Transition Temperature (Tg) (°C)—First Heating	Melting Temperature (Tm) (°C)—First Heating	Crystallization (Tc) (°C)	Cold Crystallization (Tcc) (°C)	Cold Crystallization Enthalpy (Tcc) (J/g)	Melting Enthalpy (Hm) (J/g)	Crystallinity (%)
PEEK	155.8	324.9	301.9	-	-	42.9	33
8020	163.4	322.2	300	-	-	36.7	35.2
7030	167.8	322.5	299.3	-	-	32.5	35.6
6040	170	323.5	293.2	206.7	6.6	28.3	27.8
5050	175.1	322	285.2	231.2	20.7	26.3	8.7
4060	184.9	318	278.5	227.6	13.8	18.4	8.9
2080	193.2	317.2	-	247.5	11.2	11.9	2.7
PEI	209.6	-	-	-	-	-	-

**Table 4:** First heating thermal events of 3D-printed PEEK, PEI, and their blends.

Blend	Glass Transition Temperature (Tg) (°C)—First Heating	Melting Temperature (Tm) (°C)—First Heating	Crystallization (Tc) (°C)	Cold Crystallization (Tcc) (°C)	Cold Crystallization Enthalpy (Tcc) (J/g)	Melting Enthalpy (Hm) (J/g)	Crystallinity (%)
PEEK	153.6	324.9	301.9	-	-	41.8	32
8020	163.9	324.8	299.9	-	-	36.8	35
7030	164.5	324.2	299	194.2	7.6	31.9	26.7
6040	168.3	323.2	293.6	213.3	19.5	27.5	10.3
5050	176.7	322.5	285.2	225.1	18.5	24.6	9
4060	181.6	318.8	278.3	230.9	15.1	18.3	6
2080	196.5	318	-	254.2	10.2	11.4	4
PEI	215	-	-	-	-	-	-

**Table 5:** Ultimate tensile strength (UTS) in MPa; Young’s modulus in (MPa); tensile strain in %; max force applied (KN) of PEEK, PEI, and blends (80/20, 70/30, 60/40, 50/50, 40/60, 20/80). The ultimate tensile strength, Young’s modulus, and tensile strain are reported as the mean ± standard deviation (where N = 5).

	PEEK	80/20	70/30	60/40	50/50	40/60	20/80	PEI
UTS	74.5 ± 4.04	75.6 ± 2.4	70.4 ± 6.4	70.9 ± 2.4	58.4 ± 6.2	82.6 ± 5	80.3 ± 5.8	94.5 ± 5.97
Modulus	1428 ± 41.8	1338 ± 40.9	1198 ± 65.9	1112 ± 38.3	1039 ± 78.4	1280 ± 43.5	1238 ± 36.7	1342 ± 32.2
Strain%	8.22 ± 1.32	9.42 ± 1.02	10.47 ± 1.24	10.75 ± 0.25	9.47 ± 1.35	11.9 ± 1.21	11.6 ± 1.49	11.8 ± 1.42
Max force (KN)	0.64	0.62	0.63	0.58	0.52	0.70	0.72	0.82

## Data Availability

The raw data supporting the conclusions of this article will be made available by the authors on request.

## Abbreviations

The following abbreviations are used in this manuscript:

PEEK	Polyether ether ketone
PEI	Polyetherimide
FFF	Fused filament fabrication
DSC	Differential scanning colorimetry
XRD	X-ray diffraction
SEM	Scanning electron microscopy
AM	Additive manufacturing
HPP	High-Performance polymer
FTIR	Fourier transform infrared
ATR	Attenuated total reflectance
Tm	Melting temperature
Tg	Glass transition temperature
Tc	Crystallization
Tcc	Cold crystallization
Xc	Crystallinity
UTS	Ultimate tensile strength
